# Organ Transplantation in Iran before and after Istanbul Declaration, 2008

**Published:** 2011-02-01

**Authors:** A. Nobakht Haghighi, B. Broumand, I. Fazel

**Affiliations:** 1 *Professor of Medicine and Nephrology, Shahid Beheshty University of Medical Sciences*; 2 *Professor of Medicine and Nephrology, Iran University of Medical Sciences*; 3 *Professor of Surgery Shahid Beheshty University of Medical Sciences, Tehran, Iran*

Renal transplantation in Iran dates back to 1967, when the first transplantation was carried out by Dr. Mohammad Sanadizadeh in Shiraz University, then one of the major and most advanced medical universities in Iran. During the next 12 years, the period prior to Islamic revolution, sporadic cases were transplanted, but despite the high expenses and cultural and language barrier, most of the 114 transplantations performed in this period, were carried out abroad, especially in the United Kingdom.

There was no established transplantation program in Iran and those who were transplanted were mostly done on the bases of competition and rivalry, using imported organs from Eurotransplant organ sharing network with sizable expense and guarded quality.

The limited renal transplantation activities were completely ceased two years after Islamic revolution in 1979, and qualified transplantation surgeons left the country. Iran was suffering from the post-revolution convulsive social status and a cruel devastating imposed war. All the limited resources spent on war, as well as medical manpower and hospital beds. Due to alarming shortage of hemodialysis facilities and equipment, reports of significant number of death end-stage renal disease (ESRD) patients became a daily incident.

In that situation, any suggestion for establishing a renal transplantation program was considered untimely and luxurious, even by medical professionals. Nevertheless, renal transplantation was the most promising solution to remedy the disastrous outcome of ESRD patients, and proved to be the best available mode of therapy.

Despite the grim circumstances, the first kidney transplantation after a temporary halt, was carried out in 1984 in Tehran with minimal facilities and support, but excellent outcome [1]. The organ was donated by a brother to his sister. Fifty such transplants, all from live related donors, were carried out in various setups, including charity, teaching and private hospitals in order to examine the different possibilities. This ultimately led to the development of the National plan for renal transplantation, which was formally established in a hospital dedicated to nephrology and urology patients. The hospital was university-affiliated and a mulitdisciplinary team was assigned to manage and govern the program. The excellent success rate, low cost and remarkable outcome encouraged every patient to join the waiting list for transplantation. Soon thereafter, there were ever increasing number of patients with the suitable family donor.

The cadaver organ was not available and public acceptance required long period of preparation and education. In addition, the brain death concept needed religious and legal approval. In 1989, a *fatwa* (religious approval) was issued by late Imam Khomeini at the request of one of the authors (IF), the founder of renal transplantation activity and program after Iranian revolution.

The *fatwa*, the first one in Shiath sect, confirmed the validity of brain death from religious standpoint. This was a giant step forward, but since the legal courts need valid legislation to rule, the legal approval was vitally important. After two rejections, the brain death law was finally approved by the parliament in 2000, after which time cadaver organ harvest and transplantation has become a safe practice for medical professionals. The strict guidelines for the diagnosis of brain death by multispecialty medical experts, was conveyed by the Ministry of Health and Medical Education and an active campaign for public preparation and awareness started to function. As a result, the number of cadaver donors has been steadily increasing with a more rapid pace each year. Indeed, last year 401 kidney transplants were performed from cadaver; in the first six months of 2010, this number increased to 310 transplants and we expect to have 620 in 12 months.

To prevent transplant tourism, in 1992 the high council of organ transplantation in the Ministry of Health and Medical Education ruled the forbidance of foreign nationals to be transplanted in Iran, unless they present a live donor of their own nationality. Since April 2010, following Istanbul Declaration, and to respect its contents, kidney transplantation of foreign individuals was completely removed from transplantation activities in Iran.

Despite the untiring efforts to encourage and promote cadaver organ donation, still the large gap between available organs and demand, makes the unrelated donation an alternative choice [2]. To support the donors and recipients, a state-regulated system was initiated and a reward was offered to the volunteer donors following the transplant procedure. Although this reward was acceptable at the beginning, year by year it gradually lost its real value due to inflation each year and situation evolved to present day, where the additional demand is compensated by the recipient [3].

In order to regulate this new situation and to prevent transplant tourism and commercialism, and with regard to the ethical and social importance of the issue, the Iranian Society for Organ Transplantation presented the case to the committee on Bioethics at the Academy of Medical Sciences of IR Iran. In addition to medical specialists the committee was membered by legal experts, philosophers, sociologist, psychiatrics and clergies.

Following detailed discussions in several sessions, the committee released the following document on February 4, 2008.

Since the protection of lives of human beings should be considered the most fundamental moral principal, which, however, is subjected to different cultures and social status, and to comply with up to date requirements and offer the best and advanced medical services to the patients, the Bioethics Committee of the Academy of Medical Sciences of IR Iran declared that the act of kidney donation from living related and unrelated volunteers is generally acceptable, and offering a reward as a gratitude or gift or compensation is not considered unethical and should not discourage this noble act provided:

1. The donor is truly willing to donate a kidney in right mind, and free from coercion.
2. The donor undergoes complete medical and psychological evaluation and is found to be fit for the procedure.
3. There should be no contraindication for the operation.
4. Donor should be able to get long-term medical attention.
5. The medical team has no part in process of donation.
6. Donor and recipient should be from the same nationality (tourist transplantation is forbidden).
7. No one under age of 18 and over 45 years should be accepted for donation.
8. A national committee assigned by the Ministry of Health and Medical Education with cooperation of Iranian Transplantation Society will regulate and supervise the renal transplantation centers nationwide.

Once implementation of kidney transplantation reached a satisfactory level, other forms of organ transplantation became the obvious next step. The first liver transplantation in Iran was performed by Dr. Malekhosseini in Shiraz in May of 1993 [4]. Liver transplantation expanded with a satisfactory pace in Shiraz; its rate of progression was especially boosted with the approval of brain death law and reached 210 liver transplants in year 2009 alone. Increasingly more transplant surgeons were trained in Shiraz and Tehran and new centers started organ transplantation in other major cities such as Mashhad, Isfahan, Orumieh and Kerman, and later in even smaller cities. According to the statistics released by Ministry of Health’s Office of Special Diseases and Organ Transplantation, 2141 kidney transplantations (1740 living and 401 cadaver), 210 liver (34 living and 176 cadaver), 46 heart, 16 pancreas, 11 lungs, two cases of small bowel transplantations performed by Dr. Iraj Fazel in Taleghani University Hospital in Tehran, and one case of cluster transplantation by the transplant team in Shiraz were carried out in Iran in the year 2009 alone. In addition, nowadays, bone marrow and corneal transplantations are routinely performed in many hospitals throughout Iran. It is anticipated that with expansion of the deceased organ donation, the number of organ transplants will increase even further in Iran.

Another important area, the fate of live donors and state of health, was a major concern in Iran. To fulfill this important duty, a research project to establish a protective medical network chaired by Iradj Fazel, was designed under supervision of the Academy of Medical Sciences and participation of academics active in organ transplantation. This project was initiated in 2005 and its primary findings were published in 2008. This project is actively functioning and reports will appear periodically in major medical literature [5].

To respect and execute the contents of Istanbul Declaration in 2010, the general assembly of Iranian Society for Organ Transplantation unanimously ruled the prohibition of all foreign nationalities to be transplanted from their native live unrelated donor.

In conclusion, the transplantation in Iran was organized and progressed according to the need and available facilities at time. We started with living related transplantation; when we felt the need, living unrelated transplantation was also added to the program. Finally, we were successful in expanding deceased donor program and use of other organ for helping those patients who need organ transplantation. As we noticed, occasionally there are misuses of the facilities in Iran for transplantation tourism, thus, we managed to prevent this process in order to help Istanbul Declaration to fight transplantation tourism and commercialism.
